# Eating Patterns in Patients with Compensated Cirrhosis: A Case-Control Study

**DOI:** 10.3390/nu10010060

**Published:** 2018-01-10

**Authors:** Camille Buscail, Valérie Bourcier, Léopold K. Fezeu, Dominique Roulot, Séverine Brulé, Zahia Ben-Abdesselam, Carole Cagnot, Serge Hercberg, Pierre Nahon, Nathalie Ganne-Carrié, Chantal Julia

**Affiliations:** 1 Nutritionnal Epidemiology Research Team (EREN), Paris 13 University, UMR U1153 Inserm, Inra U1125, Cnam, Center of Reasearch in Epidemiology and Biostatistics (CRESS) Sorbonne Paris Cité, 75004 Paris, France; l.fezeu@eren.smbh.univ-paris13.fr (L.K.F.); s.hercberg@eren.smbh.univ-paris13.fr (S.H.); c.julia@eren.smbh.univ-paris13.fr (C.J.); 2 Public Health Department, University Hospitals of Paris Seine-Saint-Denis, APHP, Avicenne Hospital, 93000 Bobigny, France; 3 Hepatology Department, University Hospitals of Paris Seine-Saint-Denis, APHP, Jean Verdier Hospital, 93140 Bondy, France; valerie.bourcier@aphp.fr (V.B.); mec.hge.jvr@aphp.fr (S.B.); pierre.nahon@aphp.fr (P.N.); nathalie.ganne@aphp.fr (N.G.-C.); 4 Hepatology Unit, University Hospitals of Paris Seine-Saint-Denis, APHP, Avicenne Hospital, 93000 Bobigny, France; dominique.roulot@aphp.fr (D.R.); zahia.ben-abdesselam@aphp.fr (Z.B.-A.); 5 Formation and Research Unit in Health Medicine and Human Biology, Paris 13 University, Sorbonne Paris Cité, 93430 Paris, France; 6 Hepatitis Department ANRS (France Recherche Nord & Sud, SIDA-VIH-Hépatites), 75013 Paris, France; carole.cagnot@inserm.fr; 7 UMR 1162 Inserm, Universités Paris 5, Paris 7 and Paris 13, 75006 Paris, France

**Keywords:** liver cirrhosis, alcoholic cirrhosis, viral cirrhosis, dietary intakes, nutritional status

## Abstract

Background: There is growing evidence suggesting that maintaining an adequate nutritional status for patients with liver cirrhosis (LC) is relevant to prevent complications. The present study aimed to describe dietary behaviours of patients with compensated and non-complicated LC and comparing them with those of subjects from the general population. Methods: In this case-control study, patients were volunteers enrolled in the ALICIR (ALImentation et CIRrhose) study, an observational survey nested in two French prospective cohorts of patients with biopsy-proven compensated cirrhosis related either to excessive alcohol consumption (CIRRAL) or to hepatitis B or C virus infection (CirVir). Controls were selected from the NutriNet-Santé cohort. Dietary data were collected through a semi quantitative food frequency questionnaire. Dietary and nutritional data were compared using multi-adjusted paired Student’s tests. Results: Between June 2014 and February 2016, 174 patients of CirVir (*N* = 97) or CIRRAL (*N* = 77) were matched with 348 controls from the NutriNet-Santé cohort, according to gender, age, BMI and educational level. Compared to controls, patients (mean ± SD) consumed more sodas (236.0 ± 29.8 mL vs. 83.0 ± 33.0 mL) and water (1787.6 ± 80.6 mL vs. 933.6 ± 85.3 mL), and lower amounts of salty snacks (4.2 ± 1.42 g vs. 9.0 ± 1.6 g) and alcoholic beverages (71.8 ± 23.4 g vs. 151.2 ± 25.9 g), with all *p* values < 0.0001. Dietary behaviours differed according to LC aetiology. Conclusions: Dietary behaviour of patients significantly differed from subjects from the general population.

## 1. Introduction

There is growing evidence suggesting that maintaining an adequate and balanced nutritional status for patients with liver cirrhosis (LC) is of importance. Associations between nutritional factors and long-term complications of LC have been identified, mainly in patients with decompensated forms or marked liver failure. For instance, protein-energy malnutrition (PEM) has been suggested to result in a greater risk of more severe complications such as ascites, encephalopathy, and infections. PEM has also been identified as an independent risk factor for death in patients with LC, regardless of the aetiology [1,2,3,4,5]. Moreover, long-term high protein and energy intakes resulting in overweight and insulin resistance could hasten the progression of the disease [6,7]. Similar results were shown for higher dietary cholesterol intakes [8]. More recently, Berzigotti and colleagues have shown that moderate physical activity could lead to the reduction of portal hypertension and, therefore, to a reduced risk of complications [9]. However, the dietary components involved as independent risk or protection factors of complication or mortality in LC remain largely uncertain. A few studies have focused on the dietary behaviour and lifestyle data of patients with compensated cirrhosis without significant liver failure and/or portal hypertension. They showed heterogeneous results, with no clear difference between patients and controls. However, most of them were performed in Asia, on a reduced number of patients [10,11,12,13]. Assessing dietary behaviour of LC patients during the early phase of the disease, therefore, appears essential to increase the knowledge in this domain among western populations, and to identify the role of nutritional factors in disease progression. The ALICIR (ALImentation et CIRrhose) longitudinal study was set-up to assess the dietary behaviours, lifestyle (including physical activity) and exposure to environmental factors among patients with compensated viral or alcoholic cirrhosis, and relate them to the occurrence of subsequent complications, in particular hepatocellular carcinoma (HCC). All patients have been enrolled and are ongoing close follow-up to reach critical number of events. The present cross-sectional study aimed at describing dietary behaviours of patients with compensated and non-complicated cirrhosis and comparing them with those of subjects from the general population.

## 2. Materials and Methods

### 2.1. Design of the Study

This study is based on a case-control design. The cases were recruited in two tertiary French liver units involved in the ALICIR study: Avicenne and Jean Verdier hospitals. Both these university institutions are located in the Seine-Saint-Denis district (Northeast Paris suburbs).

### 2.2. Study Population

**Figure 1:**
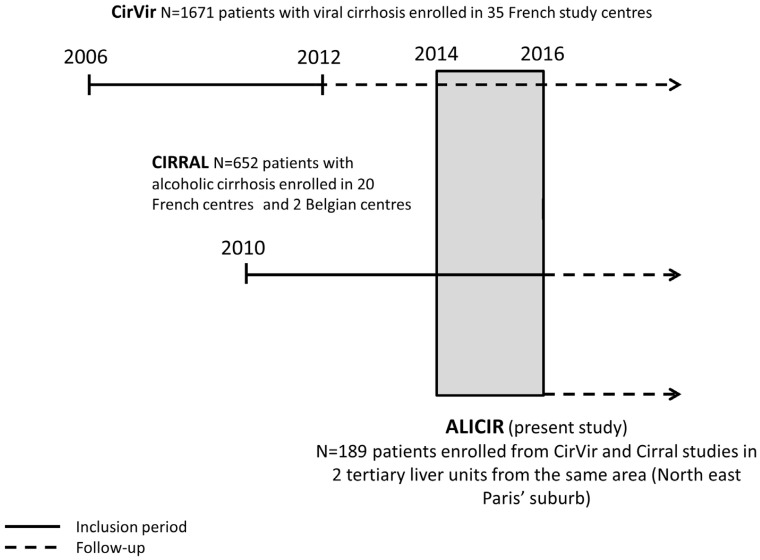
Description of patient selection.

The study population included all patients participating in the ALICIR study. The objective of the ALICIR study is to describe dietary, lifestyle, and environmental factors in LC patients and to relate them to LC complications, in particular HCC. The ALICIR study is a longitudinal cohort study nested in two French prospective cohorts of patients with cirrhosis: the ANRS CO12 CirVir and CIRRAL (Figure 1) cohorts.

The CirVir cohort included 1671 patients with histologically proven viral compensated cirrhosis, from 35 clinical centres dedicated to hepatic diseases management from March 2006 to December 2012. Excessive alcohol consumption was not an exclusion criterion. Patients included in this study receive a prospective follow-up according to French and international guidelines. This study has been fully described elsewhere [14].

The CIRRAL cohort was initiated in 2010 and included 652 patients with alcohol-related compensated cirrhosis, histologically proven, with or without HIV coinfection but without HBV or HCV infection from 22 clinical centres dedicated to hepatic diseases (ClinicalTrials.gov: number NCT01213927). Details about this study have been previously described [15].

The ALICIR study included patients enrolled in CirVir or CIRRAL between June 2014 and February 2016 in 2 tertiary liver units from the same area (north-east Paris’ suburb, University Paris 13). All patients from the CirVir or CIRRAL cohorts, who came at these hospitals for an inclusion or a follow-up visit during that period of time, and matching inclusion/exclusion ALICIR criteria, were proposed to be included in the ALICIR study. Inclusion criteria for the ALICIR study were the following: (i) included and followed-up in CirVir or CIRRAL; (ii) no detectable HCC within 90 days prior to the inclusion; (iii) signed free and informed consent. Exclusion criteria were: (i) no social insurance; (ii) lack of French-language skills; (iii) any episode of hepatic decompensation during the timeframe elapsing between CIRRAL or CirVir and ALICIR inclusion.

Controls were selected from the NutriNet-Santé Cohort (two controls were included for one patient from ALICIR). The NutriNet-Santé study is a web-based prospective observational cohort. Details on this study have been previously described [16]. Briefly, the NutriNet-Santé study aims at investigating the dietary behaviours of subjects from the general population, and their relationships with health. The inclusion of subjects aged over 18 years started in France in May 2009 and still ongoing with more than 158,000 subjects enrolled at the time of the study. At baseline, participants completed self-administered questionnaires about socio-economic, lifestyle, health status, diet, physical activity, and anthropometrics data. This set of questionnaires is repeated yearly. Moreover, during follow-up, additional questionnaires are regularly proposed on various subjects pertaining to the investigation of determinants of dietary pattern on health.

### 2.3. Ethics

The ALICIR study was approved by the French Advisory Committee for Data Processing in Health Research of the French Ministry of Health and Medical Research (CCTIRS) (file No. 13.501) and the Commission Nationale de l’Informatique et des Libertés (CNIL) (No. DR-2014-219). All patients from ALICIR study gave written informed consent before inclusion. The CirVir protocol obtained approval from the ethics committee (Comité de Protection des Personnes, Aulnay-sous-Bois, France) and conformed to the ethical guidelines of the 1975 Declaration of Helsinki. The full CirVir protocol is available via the ANRS website [17]. All patients gave written informed consent to participate in the cohort. The NutriNet-Santé Study is set in accordance with the declaration of Helsinki and was approved by the institute Review Board of the French Institute for Health and Medical Research (00000388FWA00005831) and the CNIL (No. 908450 and 909216). All participants provided an electronic informed consent.

### 2.4. Data Collection

#### 2.4.1. Dietary Data

At inclusion in ALICIR, a semi-quantitative food frequency questionnaire validated for the French population [18] was administered to patients by a trained dietician (about 1 h in duration). The questionnaire included 240 food and beverage items, categorized in 24 food groups. For each group portion size are estimated using either usual containers (for example spoon or standard unit as yogurt) or a set of validated color photographs (for example, three different plates with various portions of pastas are used for the assessment of starches consumption). This questionnaire is based on the food frequency questionnaire validated in the French cohort SU.VI.MAX [19]. Unless they needed specific dietary advices related to other chronic conditions such as diabetes, obesity or heart failure, patients did not benefit from a dietary counselling before the completion of the questionnaire. Dietary data for controls were collected using an identical questionnaire, also self-administered online, sent to each participant eight months after inclusion in the NutriNet-Santé study. Both cases and control were asked to recall their intake over the previous 12 months.

#### 2.4.2. Covariates

Data about lifestyle (marital status, educational level, socioeconomic status, smoking status, physical activity level, weight history, native country, and length of stay in France for patients born in foreign countries); nutritional behaviours (alcohol consumption, and food supply), environmental exposure, and medical history (such as high blood pressure, diabetes); and current treatment were collected at inclusion in both studies using similar questionnaires.

#### 2.4.3. Patients’ Characteristics

At inclusion in the ALICIR study, patients completed self-reported questionnaires on sociodemographic data (age, gender, marital status, educational level, professional status, living place, country of birth), family medical history of liver cancer, smoking status and cannabis use, weight and weight change over the last five years. Physical activity level was assessed using the International Physical Activity Questionnaire (IPAQ) at baseline, and the Metabolic Equivalent of Task (MET) scores based on the classification of Ainsworth [20] were used to calculate a total MET for each volunteer. Subjects are classified according to their total level of physical activity (1: subjects highly physically active; 2: subjects with intermediate level of total physical activity; 3: subjects with low level of total physical activity) according to the IPAQ guidelines [20].

#### 2.4.4. Controls’ Characteristics

At baseline, information on age, gender, body mass index (BMI) (normal/overweight or obese), smoking status (current smoker/former smoker/nonsmoker), marital status (single/cohabiting), monthly income level (<1200 € per consumer unit (c.u.)/1200–2300 € per c.u./>2300 € per c.u.) [21] and educational level (no diploma or primary studies/secondary studies or higher educational level) were collected by self-administered questionnaire. Physical activity level was assessed using International Physical Activity Questionnaire (IPAQ) at baseline [20].

### 2.5. Statistical Analysis

Controls (two for one case) were selected among participants in the NutriNet-Santé study residing in the same geographical region as the two sites of inclusion in the ALICIR study. Moreover, cases and controls were matched according to their gender, age (five years), BMI (<25 kg/m^2^, [25–30), ≥30 kg/m^2^), and educational level (no diploma and primary, secondary school, superior). The comparison of sociodemographic and lifestyle data between case and control groups was performed using chi-square tests. Comparison of food group consumption and nutrients intake between cases and controls was realized, using ANCOVA tests, and was controlled at least for occupational level, marital status, smoking status, physical activity. Analyses were also controlled for total energy intake for alcohol. Comparison of water consumption was further controlled for diabetes status and diuretic treatment. Data on intakes of saturated fatty acids (SFA), monounsaturated fatty acids (MUFA), polyunsaturated fatty acids (PUFA), beta-carotene, and vitamins A, B12, C, and E were first log transformed to comply with normal distribution. Vitamins intakes were compared between cases and controls according to the prevalence of nutrient inadequacy regarding the French estimated average requirements (EAR) (i.e., the proportion of subjects with reported intakes below the EAR). This prevalence represents an unbiased estimate of the proportion of subjects whose intakes are below their respective requirements [22]. We relied upon the French references updated in 2016 to compute the prevalence of nutrient inadequacy in both groups [23]. These proportions were then compared between cases and controls using chi-square tests. All tests of significance were two-sided and the type I error was set at 10^−3^, given the high number of tests performed. All analyses were carried out using SAS software (version 9.4; SAS Institute, Inc., Cary, NC, USA) [24].

## 3. Results

**Figure 2:**
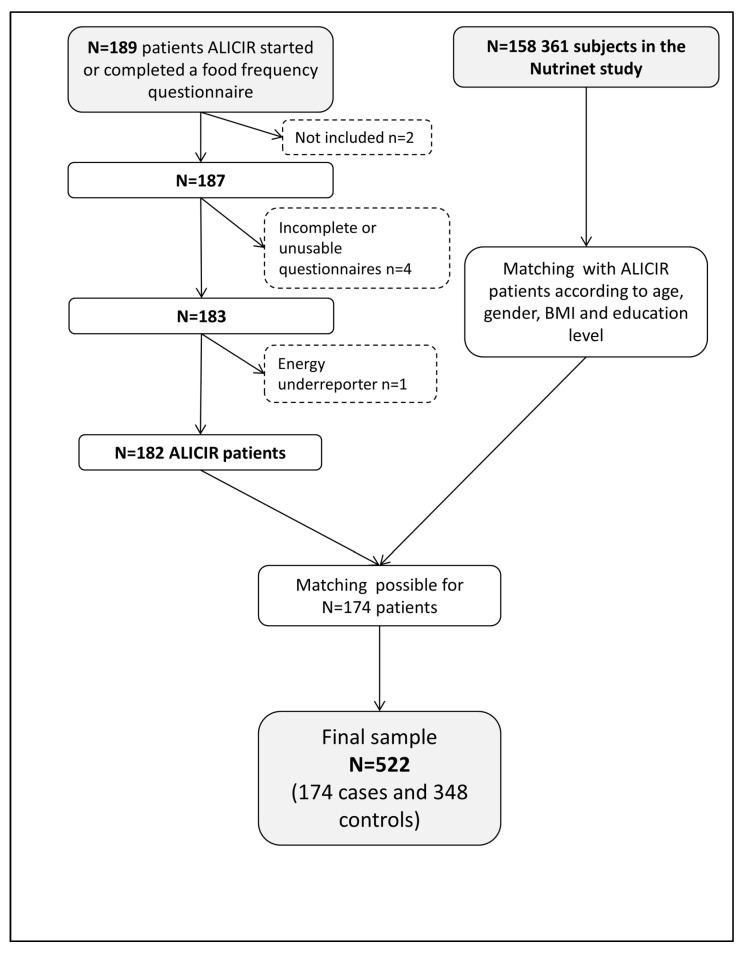
Flowchart of the study.

Between June 2014 and February 2016, 189 patients completed (at least partially) the dietary questionnaire. Two patients were excluded because they did not meet the inclusion criteria. Four questionnaires were excluded because answers were missing for at least 10 items in the questionnaire. One patient reporting 178 kcal of daily energy intake was excluded. Finally, 182 patients included in the ALICIR study had complete dietary data available for analysis. Matching between cases and controls was available for 174 patients of the ALICIR study. Subjects for which controls could not be selected in the NutriNet-Santé study were all aged >80 years old and with low educational levels. Thus, data for 174 cases and 348 controls were used for this study (Figure 2). Patients were mainly men (72.4%) aged between 55 and 65 years (39.7%, mean age ± SD was 59.1 ± 9.7 years), with an increased BMI (62.1% over 25 kg/m^2^). Less than a half of patients were born in France (*N* = 83, 47.7%). Others patients were born in Sub-Saharan Africa (*N* = 28, 16.1%), in Europe (except France, *N* = 22, 12.6%), in Maghreb (*N* = 21, 12.1%), and in Asia or America (*N* = 20, 11.4%). Forty-nine patients (33.1%) (*N* = 27 in alcoholic LC and *N* = 22 in viral LC) were suffering from diabetes and 48 (32.4%) from high blood pressure (*N* = 18 in alcoholic LC and *N* = 30 in viral LC) (*N* = 26 missing data for the both variables). Except for the marital status for which no significant difference was observed between patients and controls, the cases from ALICIR were more likely to be on disability leave from work, more often smokers and had a lower physical activity level (all *p* values were < 0.0001) (Table 1). These sociodemographic differences between cases and controls were similar according to the different aetiologies of the cirrhosis (Table 2).

### 3.1. Comparison of Dietary Intakes

Overall, compared to the NutriNet-Santé study controls, patients consumed higher amounts of sodas (236.0 ± 29.8 mL vs. 83.0 ± 33.0 mL, *p* < 0.0001) and water (1787.6 ± 80.6 mL vs. 933.6 ± 85.3 mL, *p* < 0.0001). Conversely, they ate less legumes, salty snacks, vegetable fat, and drank fewer alcoholic beverages (Table 3). Dietary behaviours somewhat differed according to LC aetiology: subjects with alcoholic LC showed higher intakes in sweet products (i.e., marmalade, confectionery and honey) compared to the control group (supplemental Table S1). The patients with viral LC showed lower consumptions of legumes, processed meat, desserts, and salty snacks.

### 3.2. Comparison of Nutrients Intakes

Compared to controls, the diet of patients from the ALICIR study had lower contents of proteins, especially animal proteins, lipids (including SFA and PUFA), and alcohol. Conversely, they had higher carbohydrates and sodium, intakes (Table 4). Prevalence of inadequacy regarding EAR was significantly higher in cases for vitamins B6, C, and E. Results were similar for the two aetiologies, except for fat intakes, which were lower than in the control group for patients with viral LC, while they were higher in patients with alcoholic cirrhosis (Supplemental Table S2).

## 4. Discussion

In this case-control study, we compared food consumption and nutrient intakes between patients with a compensated and non-complicated LC and controls from the general population. The results showed that dietary and nutritional profiles of patients with LC differed from those of controls, and between the two aetiologies of LC (alcohol and virus). To the best of our knowledge, this is the first study focusing on dietary behaviour and nutritional intakes of patients with a compensated non-complicated LC performed in Europe.

In line with previous studies, our results showed that dietary behaviours of patients with LC differed from those of controls. First of all, overall dietary behaviour of patients tended to be less healthy. They had a lower consumption of legumes, vegetable fat, and soft and non-sugared beverages, and higher intakes of sodas, sauces, and desserts. Similar results were shown by Loguercio and colleagues, in a case-control study performed on 40 patients with either alcoholic or viral LC and 30 controls [25]. Compared to controls, cases ate less salty snacks and had lower intakes of alcohol. Overall, these results are consistent with nutritional guidelines for LC patients, which recommend the avoidance of alcohol consumption and moderate sodium intakes [25,26,27].

However, we also found that dietary habits differed according to the LC aetiology, especially regarding sweet products and starches, which were consumed in higher amounts in alcoholic LC patients compared to controls, and not in viral LC patients [25]. Given the potential impact of sugar intake on LC complications, these results suggest that patients may have differing levels of risk depending on the aetiology of LC.

Regarding the nutritional status, patients tended to have diets with higher intakes of carbohydrates, at the expense of protein (in particular animal protein) and lipid intakes patients compared to controls. This is in line with previous studies that showed higher prevalence of protein-energetic malnutrition in patients with cirrhosis, including in the early history of the disease [1,10,28]. Given this knowledge, the European Society for Clinical Nutrition and Metabolism (ESPEN) established specific guidelines (last update in April 2006) for patients with a liver disease and transplantation [27,29]. The recommended intakes of energy and proteins for patients with compensated and non-complicated cirrhosis are 35–40 kcal/kg/day and 1.2–1.5 g/kg/day, respectively. In our study, daily energy intakes were lower than this bound in both alcoholic and viral cirrhosis (30.1 ± 1.8 kcal/kg/day and 26.8 ± 1.6 kcal/kg/day respectively). The daily amounts of protein intakes reached the recommendations for patients with an alcoholic cirrhosis (1.3 ± 0.1), but not for patients with a viral cirrhosis (1.1 ± 0.1). Therefore, though our study did not show significant differences between cases and controls for energy intakes, the specific requirements for energy intakes in patients were not reached. Moreover, the cases in our study showed higher sodium intakes than controls, and intakes over 3 g/day, while the ESPEN guidelines recommend a salt-restricted diet (2–3 g of salt, corresponding to 5–7 g of salt per day) especially in the case of hydro sodium retention. However, the patients of ALICIR have a clinical presentation of compensated and non-complicated cirrhosis, thus, with the exception of specific diets related to comorbidities, such as heart failure, patients were not usually encouraged to adopt a salt-free or salt-restricted diet. Then, it is expected that the behavior of patients regarding the salt consumption is similar to those of the general population. The difference we showed between cases and controls could partly be due to increased intakes of sauces, cereal bread, and processed meat in patients, which are the main sources of salt in the French diet [30]. Finally, the assessment of sodium could be less accurate for volunteers. Indeed, web-based self-administered questionnaires tend to minimize the salt consumption [31,32].

We found high water—and soda—consumption in cases, rising up to a mean 1.8 L ± 81 mL per day compared to 933 ± 85 mL per day in controls. The relation between cirrhosis and renal function is not well understood, and is subject to extensive research. However, several studies have highlighted an internal dysregulation of the renin system in patients with stable LC [33,34]. The elevated beverage intakes in patients may, therefore, be consistent with preclinical renal dysfunction symptoms, even at this early stage of the disease. An alternative explanation would be related to a modification of dietary behaviour, with the replacement of other beverages, like coffee, tea or, more specifically, alcoholic beverage in patients with alcoholic LC by water. To our knowledge, this is the first study to identify this specific pattern of beverage consumption in LC patients. Further studies are required to better understand the determinants of such consumption, and investigate the potential relationship between liver and renal malfunctions.

This study has numerous strengths that ensure reliable results. First, patients were unambiguously identified with biopsy-proven cirrhosis and were selected with stringent criteria regarding liver function. Second, we were able to match patients with a large number of controls from the general population, using a large set of matching criteria limiting the differences between patients and controls. Third, while most studies in LC patients focused on proteins and energy intakes, to investigate protein-energy malnutrition, we were able to compare a wider spectrum of dietary data. Finally, the food frequency questionnaire that was used in both patients and controls to assess dietary behaviour, was previously validated in a French population, and showed good validity and reproducibility [18].

This study, however, presents some limitations. Though we used a large set of variables to match cases and controls, some specific aspects that impact dietary behavior were not taken into account. Indeed, cases included a large share of patients with having a history of migration (North and sub-Saharan Africa for almost two thirds of the patients), which could, in part, explain the differences observed, since dietary behavior is largely influenced by socio-cultural background [35]. We were not able to compare ethnic backgrounds between cases and controls, since this information is not collected in the Nutrinet Santé study (this is due to the French legislation regarding personal data collection). However, dietary habits have been shown to be altered following immigration, and the combination of a traditional diet with items from the western diet is frequently observed [36]. Moreover, participants included in the NutriNet-Santé cohort are adult volunteers and are, therefore, more likely to be interested in nutrition and, therefore, adopt healthier dietary and lifestyle behaviors than the general population, which could have widened the observed differences between cases and controls [37]. This assumption was supported by the differences we showed between cases and controls regarding smoking status, occupational status, and physical activity level (Table 1 and Table 2). Thus, an alternative method to conduct the study could be the comparison of patients with their healthy relatives, so that the ethical or familiar peculiarity in diet habits could be minimized. Finally, the different method used for the completion of the food questionnaires (i.e., web self-administered versus hetero-administration by a dietician) could have partly impacted the dietary and/or nutritional information. Yet, web-based self-administered Food Frequency Questionnaire (FFQ) have shown their validity and reproducibility in comparison with the same questionnaire using a hetero-administration [38].

## 5. Conclusions

In this case-control study, patients with non-complicated and compensated LC showed overall less healthy dietary habits compared to controls, which somewhat differed according to the origin of the cirrhosis. Additionally, patients did not meet the European nutritional guidelines regarding protein, energy, and sodium intakes, especially in the case of viral cirrhosis. Long-term follow-up of these patients among the ALICIR study should contribute to increasing the knowledge of how nutritional and dietary factors influence the progression of the disease, especially regarding the occurrence of HCC.

## Figures and Tables

**Table 1:** Comparison of sociodemographic characteristics for cases and controls (*N* = 522).

	ALICIR		NutriNet		p *
	*N*	*%*	*N*	*%*	
	174		348		
**Gender**					
Male	126	72.4	252	72.4	1.00
Female	48	27.6	96	27.6	
**Age (years)**					
≤45	12	6.9	24	6.9	
45–54.9	41	23.6	82	23.6	
55–64.9	69	39.7	138	39.7	1.00
65–74.9	41	23.6	82	23.6	
≥75	11	6.3	22	6.3	
**BMI (kg/m^2^)**					
<25	66	37.9	132	37.9	
[25–30)	67	38.5	134	38.5	1.00
≥30	41	23.6	82	23.6	
**Education level**					
No diploma or primary school	119	68.4	238	68.4	
Secondary	21	12.1	42	12.1	1.00
High education level	34	19.5	68	19.5	
**Marital status**					
Single	57	32.8	91	26.1	0.11
Cohabiting	117	67.2	257	73.8	
**Professional status**					
Working	68	39.1	141	40.5	
Unemployed	88	50.6	202	58.0	<0.0001
Sick leave	18	10.3	5	1.4	
**Smoking status**					
Former or non-smoker	123	70.7	313	89.9	<0.0001
Current smoker	51	29.3	35	10.1	
**Physical activity level**					
High	25	14.4	147	42.2	
Moderate	88	50.6	121	34.8	<0.0001
Low	47	27.0	80	23.0	
Missing	14	8.0	0	0.00	

Abbreviations: BMI: Body Mass Index. * Chi-square tests were performed.

**Table 2:** Comparison of sociodemographic characteristics for cases and controls stratified on the aetiology of cirrhosis (*N* = 522).

	ALICIR		NutriNet			ALICIR		NutriNet		
	Alcoholic Cirrhosis					Viral Cirrhosis				
	*N*	*%*	*N*	*%*	p *	*N*	*%*	*N*	*%*	p *
	77		154			97		194		
**Gender**										
Men	58	75.3	116	75.3	1.00	68	70.1	136	70.1	1.00
Women	19	24.6	38	24.7		29	29.9	58	29.9	
**Age**										
≤45 years	1	1.3	2	1.3		11	11.3	22	11.3	
45–55 years	18	23.4	36	23.4		23	23.7	46	23.7	
55–65 years	31	40.3	42	40.3	1.00	38	39.2	76	39.2	1.00
65–75 years	24	31.2	48	31.2		17	1.5	34	17.5	
>75 years	3	3.9	6	3.9		8	8.2	16	8.2	
**BMI**										
<25	25	32.4	50	32.5		41	42.3	82	42.3	
[25–30)	27	35.1	54	35.1	1.00	40	41.2	80	41.2	1.00
≥30	25	32.5	50	32.5		16	16.5	32	16.5	
**Education level**										
No diploma or primary school	55	71.4	110	71.4		64	66.0	128	66.0	
Secondary	7	9.1	14	9.1	1.00	14	14.4	28	14.4	1.00
High education level	15	19.5	30	19.5		19	19.6	38	19.6	
**Marital status**										
Single	28	36.4	40	26.0	0.10	29	29.9	51	26.3	0.52
Cohabiting	49	63.6	114	74.0		68	70.1	143	73.7	
**Professional status**										
Employed	20	26.0	52	33.8		48	49.5	89	45.9	
Unemployed	47	61.0	99	64.3	0.002	41	42.3	103	53.1	0.003
Sick leave	10	13.0	3	1.9		8	8.2	2	1.0	
**Smoking status**										
Former or non smoker	47	61.0	140	90.9	<0.0001	76	78.3	173	89.2	0.01
Current smoker	30	39.0	14	9.19		21	21.6	21	10.8	
**Physical activity level**										
High	9	11.7	56	36.4		16	16.5	91	46.9	
Moderate	41	53.2	60	39.0	<0.0001	47	48.4	61	31.4	<0.0001
Low	20	26.0	38	24.7		27	27.8	42	21.6	
Missing	7	9.1	0			7	7.2	0		

Abbreviations: BMI: Body Mass Index. * Paired Chi-square tests were performed.

**Table 3:** Comparison of adjusted dietary intakes between controls and cases (*N* = 522).

	ALICIR Mean (SD)	NutriNet Mean (SD)	*p* *
*N*	174	348	
Fruits (g/day)	205.9 (25.1)	258.1 (27.7)	0.18
Vegetables (g/day)	276.9 (22.3)	317.2 (24.7)	0.12
Cereal bread (g/day)	142.2 (8.1)	125.225 (9.0)	0.09
Potatoes (g/day)	35.1 (2.9)	29.1 (3.2)	0.03
Pasta, rice, semolina (g/day)	117.2 (9.9)	98.8 (11.1)	0.01
**Legumes (g/day)**	**14.6 (2.6)**	**26.0 (2.9)**	**<0.0001**
Milk (g/day)	142.4 (21.4)	116.0 (23.6)	<0.01
Dairy products (g/day)	159.9 (18.0)	193.1 (19.8)	0.02
Cheese (g/day)	36.3 (5.4)	50.8 (5.9)	<0.01
Fish and seafood (g/day)	39.6 (5.4)	50.9 (6.0)	0.03
Meat (g/day)	101.4 (8.0)	99.0 (8.9)	0.88
Poultry (g/day)	27.8 (2.9)	21.1 (3.2)	<0.01
Organ meat (g/day)	6.0 (1.0)	7.4 (1.1)	0.03
Eggs (g/day)	15.9 (1.4)	12.7 (1.6)	0.05
Processed meat (g/day)	9.1 (1.8)	8.8 (2.0)	0.06
Desserts (g/day)	23.2 (4.8)	15.7 (5.3)	0.28
Marmelade, confectionery and honey (g/day)	29.4 (2.3)	23.0 (2.5)	<0.01
Cakes and cookies (g/day)	28.0 (3.3)	28.4 (3.7)	0.05
**Salty snacks (g/day)**	**4.2 (1.4)**	**9.0 (1.6)**	**<0.0001**
**Sauces (g/day** **)**	**18.4 (1.2)**	**9.4 (1.3)**	**<0.0001**
Animal fat (g/day)	4.5 (0.8)	6.3 (0.8)	0.48
**Vegetable fat (g/day)**	**14.2 (2.1)**	**20.0 (2.3)**	**<0.001**
**Water (g/day)**	**1787.6 (80.6)**	**933.6 (85.3)**	**<0.0001**
Soft beer (g/day)	9.0 (6.5)	9.0 (7.2)	0.86
**Sodas (g/day** **)**	**236.0 (29.8)**	**83.0 (33.0)**	**0.0001**
**Alcoholic beverages (g/day** **)**	**71.8 (23.4)**	**151.2 (25.9)**	**<0.0001**
Coffee (g/day)	131.3 (18.6)	178.8 (20.5)	<0.01
Tea (g/day)	101.0 (26.7)	140.1 (29.5)	0.03
Soft and non-sugared beverages (g/day)	59.5 (16.0)	86.1 (17.7)	<0.01

* ANCOVA tests adjusted for: marital status, professional status, smoking status, and physical activity. Water intakes were also adjusted for diabetes status and diuretic treatment.

**Table 4:** Comparison of adjusted nutrient intakes between controls and cases (*N* = 522).

	ALICIR	NutriNet	*p* *
Total energy intake (kcal/day)	2104.4 (84.4)	2202.8 (93.3)	0.23
Proteins (%TEI)	17.7 (0.4)	18.5 (0.5)	0.03
Animal proteins (%TEI)	12.6 (0.47)	13.3 (0.5)	0.11
Vegetable proteins (%TEI)	5.1 (0.2)	5.2 (0.2)	0.41
**Carbohydrates (%TEI)**	**45.8 (0.9)**	**38.4 (1.0)**	**<0.0001**
**Simple carbohydrates (%TEI)**	**21.6 (0.7)**	**17.9 (0.8)**	**<0.0001**
**Lipids (%TEI)**	**34.8 (0.8)**	**38.1 (0.9)**	**<0.0001**
SFA (%TEI)	13.3 (0.4)	14.5 (0.4)	<0.01
MUFA (%TEI)	13.4 (0.4)	14.5 (0.4)	<0.01
PUFA (%TEI)	5.6 (0.3)	6.2 (0.3)	<0.01
**Alcohol (%TEI)**	**1.7 (0.5)**	**4.5 (0.6)**	**<0.0001**
**Sodium (mg/day)**	**3289.8 (126.8)**	**2879.3 (140.2)**	**<0.0001**
*Prevalence of inadequacy regarding Estimated Average Requirements (EAR, N, %)* ^†^			
Vitamin A	99 (56.9%)	170 (50.6%	0.17
Beta-caroten	78 (44.8%)	129 (37.1%)	0.09
Vitamin B1	109 (62.6%)	183 (52.6%)	0.03
Vitamin B6	79 (45.4%)	108 (31.0%)	<0.01
Vitamin B12	41 (23.6%)	54 (15.5%)	0.02
Vitamin C	69 (39.7%)	103 (29.6%)	0.02
Vitamin E	77 (44.2%)	114 (32.8%)	0.01

Abbreviations: EAR: Estimated Average Requirements, TEI: Total Energy Intake. * ANCOVA tests adjusted for marital status, professional status, smoking status and physical activity. For alcohol, tests were also adjusted for total energy intake. ^†^ Chi-square tests were performed. EAR for adult men: Vitamin A: 570 µg/day; Vitamin B1: 1.50 mg/day; Vitamin B6: 1.80 mg/day; Vitamin B12: 4.00 µg/day; Vitamin C: 90.0 mg/day; Vitamin E: 10.50 mg/day. EAR for adult women: Vitamin A: 490 µg/day; Vitamin B1: 1.20 mg/day; Vitamin B6: 1.50 mg/day; Vitamin B12: 4.00 µg/day; Vitamin C: 90.0 mg/day; Vitamin E: 9.90 mg/day. For beta-carotene, usual intakes among the general population were used: 3228.80 µg/day for both men and women.

## Supplementary Materials

The following are available online at www.mdpi.com/2072-6643/10/1/60/s1, Table S1: Comparison of dietary intakes stratified on the aetiology of cirrhosis (*N* = 522); Table S2: Comparison of nutrient intakes stratified on the aetiology of cirrhosis (*N* = 522).
