# Supplementary material for: Eating Patterns in Patients with Compensated Cirrhosis: A Case-Control Study
Source: Nutrients. 2018 Jan 10;10(1):60. doi: 10.3390/nu10010060 (PMC5793288; doi:10.3390/nu10010060)
Supplement: Supplementary file 1 [file nutrients-10-00060-s001.pdf]

Table S1. Comparison of dietary intakes stratified on the aetiology of cirrhosis (*N* = 522).

	**ALICIR Alcoholic cirrhosis (*N* = 77)**	**NutriNet (*N* = 154)**	***p****	**ALICIR Viral cirrhosis (*N* = 97)**	**NutriNet (*N* = 194)**	***p****
Fruits (g/day)	228.9 (39. 7)	256.7 (44.8)	0.90	190.6 (32.9)	253.5 (35.6)	0.06
Vegetables (g/day)	284.7 (29.7)	326.7 (33.5)	0.18	277.1 (33.8)	320.7 (36.5)	0.22
Cereal bread (g/day)	133.2 (11.1)	118.7 (12.5)	0.54	154.3 (12.2)	136.3 (13.1)	0.11
Potatoes (g/day)	33.0 (4.1)	26.0 (4.7)	0.02	36.5 (4.2)	32.0 (4.5)	0.47
Pasta, rice, semolina (g/day)	83.3 (11.7)	84.3 (13.3)	0.76	143.9 (15.6)	118.5 (16.8)	**0.02**
**Legumes (g/day)**	15.5 (3.6)	27.5 (4.1)	<0.01	**13.0 (3.9)**	**24.5 (4.2)**	**<0.0001**
Milk (g/day)	110.3 (27.0)	129.9 (30.4)	0.24	160.0 (33.2)	113.69 (35.9)	0.03
Dairy products (g/day)	172.1 (30.9)	199.3 (34.9)	0.36	145.3 (20.9)	183.5 (22.6)	0.01
Cheese (g/day)	39.9 (9.4)	50.3 (10.6)	0.71	36.7 (5.7)	53.8 (6.1)	<0.01
Fish and seafood (g/day)	38.5 (6.6)	51.0 (7.5)	0.05	43.5 (8.6)	53.4 (9.2)	0.35
Meat (g/day)	115.5 (12.4)	105.9 (14.0)	0.48	88.6 (10.5)	93.3 (11.4)	0.54
Poultry (g/day)	30.4 (3.8)	25.8 (4.3)	0.05	25.4 (4.4)	17.3 (4.8)	0.08
Organ meat (g/day)	6.5 (1.6)	8.5 (1.8)	0.13	5.4 (1.3)	6.8 (1.4)	0.06
Eggs (g/day)	17.3 (2.3)	13.4 (2.6)	0.13	15.0 (1.8)	12.5 (2.0)	0.20
**Processed meat (g/day)**	12.2 (2.3)	7.0 (2.5)	0.41	**6.3 (2.9)**	**10.0 (3.1)**	**<0.001**
**Desserts (g/day)**	33.7 (8.1)	5.5 (9.2)	0.05	**15.4 (5.7)**	**23.0 (6.1)**	**<0.001**
Marmelade, confectionery and honey (g/day)	32.1 (3.3)	22.9 (3.8)	<0.01	27.8 (3.2)	23.7 (3.4)	0.26
Cakes and cookies (g/day)	27.7 (4.3)	21.0 (4.9)	0.77	29.6 (5.1)	35.9 (5.5)	<0.01
**Salty snacks (g/day)**	3.9 (1.8)	7.6 (2.0)	0.01	**4.3 (2.2)**	**10.1 (2.4)**	**<0.001**
**Sauces (g/day)**	**16.8 (1.4)**	**8.3 (1.6)**	**<0.0001**	**19.5 (1.8)**	**10.8 (1.9)**	**<0.0001**
Animal fat (g/day)	4.6 (1.1)	6.9 (1.2)	0.90	3.9 (1.1)	5.4 (1.2)	0.44
**Vegetable fat (g/day)**	**15.4 (3.4)**	**22.7 (3.9)**	**0.0001**	12.6 (2.7)	18.1 (2.9)	0.09
**Water (g/day)**	**1763.3 (120.5)**	**900.8 (129.0)**	**<0.0001**	**1892.3 (110.0)**	**1032.5 (115.4)**	**<0.0001**
Soft beer (g/day)	20.7 (13.8)	25.4 (15.6)	0.35	0.9 (1.1)	0.2 (1.1)	0.72
**Sodas (g/day)**	**315.1 (49.3)**	**46.3 (55.6)**	**<0.0001**	170.4 (35.2)	98.0 (38.1)	0.28
**Alcoholic beverages (g/day)**	**102.9 (40.5)**	**134.3 (45.8)**	**<0.0001**	**49.3 (26.2)**	**153.9 (28.3)**	**<0.0001**
Coffee (g/day)	114.6 (21.6)	169.3 (24.4)	0.01	149.4 (29.9)	194.3 (32.3)	0.04
Tea (g/day)	104.8 (42.2)	180.6 (47.7)	0.04	102.5 (34.8)	103.2 (37.7)	0.36
Soft and non-sugared beverages (g/day)	74.7 (24.2)	77.4 (27.3)	0.02	37.4 (21.9)	81.9 (23.6)	0.047

* ANCOVA tests adjusted for: marital status, professional status, smoking status, physical activity, and total energy intake. Water intakes were also adjusted for diabetes and diuretic treatment. Numbers in bold are significant p values (less than 0.0001)

Table S2. Comparison of nutrient intakes stratified on the aetiology of cirrhosis (*N* = 522).

	ALICIR Alcoholic cirrhosis (*N* = 74)	NutriNet	*p**	ALICIR Viral cirrhosis (*N* = 97)	NutriNet	*p**
Total energy intake (kcal/day)	2172.57 (127.97)	2124.63 (144.49)	*0.73*	2066.85 (111.88)	2306.06 (120.90)	*0.02*
Proteins (%TEI)	18.20 (0.60)	19.39 (0.68)	*0.07*	17.29 (0.59)	17.82 (0.63)	*0.31*
Animal proteins (%TEI)	13.45 (0.68)	14.25 (0.77)	*0.27*	11.93 (0.67)	12.51 (0.73)	*0.34*
Vegetable proteins (%TEI)	4.75 (0.23)	5.15 (0.26)	*0.11*	5.35 (0.24)	5.32 (0.26)	*0.87*
**Carbohydrates (%TEI)**	**43.88 (1.36)**	**36.93 (1.53)**	***<0.0001***	**47.37 (1.27)**	**39.81 (1.37)**	***<0.0001***
**Simple carbohydrates (%TEI)**	**22.54 (1.04)**	**17.00 (1.17)**	***<0.0001***	20.83 (0.96)	18.27 (1.04)	*<0.01*
**Lipids (%TEI)**	35.80 (1.14)	38.91 (1.29)	*0.01*	**33.78 (1.16)**	**37.55 (1.25)**	***<0.001***
**SFA (%TEI)**	13.98 (0.56)	14.52 (0.63)	*0.36*	**12.71 (0.56)**	**14.47 (0.61)**	***<0.001***
MUFA (%TEI)	13.80 (0.54)	14.93 (0.61)	*0.05*	13.05 (0.62)	14.26 (0.67)	*0.03*
PUFA (%TEI)	5.54 (0.39)	6.53 (0.44)	*<0.01*	5.63 (0.35)	5.99 (0.38)	*0.17*
**Sodium (mg/day)**	**3387.98 (191.6)**	**2804.15 (216.3)**	***<0.0001***	**3265.79 (168.7)**	**3011.68 (182.30)**	***<0.0001***
**Alcohol (%TEI)†**	**2.10 (0.85)**	**4.39 (0.96)**	***<0.0001***	**1.57 (0.61)**	**4.45 (0.65)**	***<0.0001***
Prevalence of inadequacy regarding Estimated Average Requirements (EAR, N %)						
Vitamin A	39 (50.6 %)	74 (48 %)	*0.70*	60 (61.9 %)	102 (52.6 %)	*0.13*
Beta-caroten	37 (48.0 %)	63 (40.9 %)	*0.30*	41 (42.3 %)	66 (34 %)	*0.17*
**Vitamin B1**	**46 (59.7 %)**	**85 (55.2 %)**	***0.51***	**63 (64.9 %)**	**98 (50.5 %)**	***0.02***
**Vitamin B6**	**35 (45.4 %)**	**51 (33.1 %)**	***0.07***	**44 (45.4 %)**	**57 (29.4 %)**	***<0.01***
Vitamin B12	17 (22.1 %)	20 (13 %)	*0.07*	24 (24.7 %)	34 (17.5 %)	*0.15*
Vitamin C	35 (45.5 %)	52 (33.8 %)	*0.08*	34 (35 %)	51 (26.3 %)	*0.12*
Vitamin E	37 (48 %)	55 (35.7 %)	*0.07*	40 (41.2 %)	59 (30.4 %)	*0.07*

Abbreviations : EAR: Estimated Average Requirements; TEI: Total Energy Intake

* ANCOVA tests adjusted for marital status, professional status, smoking status, physical activity. † for alcohol. ANCOVA tests were also adjusted for total energy intake.

† Chi-square tests were performed. EAR for adult men: Vitamin A: 570 μg/day; Vitamin B1: 1.50 mg/day; Vitamin B6: 1.80 mg/day; Vitamin B12: 4.00 μg/day; Vitamin C: 90.0 mg/day; Vitamin E: 10.50 mg/day. EAR for adult women: Vitamin A: 490 μg/day; Vitamin B1: 1.20 mg/day; Vitamin B6: 1.50 mg/day; Vitamin B12: 4.00 μg/day; Vitamin C: 90.0 mg/day; Vitamin E: 9.90 mg/day. For beta-carotene, usual intakes among the general population were used: 3228.80 μg/day for both men and women.
